# Identification of Proteins Associated with Ovarian Cancer Chemotherapy Resistance Using MALDI-MSI

**DOI:** 10.3390/ijms26125893

**Published:** 2025-06-19

**Authors:** Tannith M. Noye, Parul Mittal, Zoe K. Price, Annie Fewster, Georgia Williams, Tara L. Pukala, Manuela Klingler-Hoffmann, Peter Hoffmann, Martin K. Oehler, Noor A. Lokman, Carmela Ricciardelli

**Affiliations:** 1 Adelaide Medical School, Robinson Research Institute, University of Adelaide, Adelaide, SA 5005, Australia; tannith.noye@adelaide.edu.au (T.M.N.); zoe.price@adelaide.edu.au (Z.K.P.); afefewster@gmail.com (A.F.); georgiakwilliams28@gmail.com (G.W.); martin.oehler@adelaide.edu.au (M.K.O.); 2 Mass Spectrometry and Proteomics Group, Clinical & Health Sciences, Centre for Pharmaceutical Innovation, University of South Australia, Adelaide, SA 5005, Australia; parul.mittal@unisa.edu.au (P.M.); manuela.klingler-hoffmann@unisa.edu.au (M.K.-H.); peter.hoffmann@unisa.edu.au (P.H.); 3 Discipline of Chemistry, School of Physics, Chemistry and Earth Sciences, University of Adelaide, Adelaide, SA 5005, Australia; tara.pukala@adelaide.edu.au; 4 Department of Gynaecological Oncology, Royal Adelaide Hospital, Adelaide, SA 5005, Australia

**Keywords:** ovarian cancer, MALDI-MSI, collagen alpha 1(XII), plectin, chemotherapy resistance

## Abstract

Ovarian cancer is the most lethal gynecological cancer. Up to 75% of cases are high-grade serous ovarian cancer (HGSOC) that have high chemosensitivity to first-line platinum-based therapies. However, 75% of patients will become chemoresistant following relapse. The underlying mechanism for developing resistance to chemotherapy in HGSOC is poorly understood. In this study, we employed Matrix-Assisted Laser Desorption/Ionization–Mass Spectrometry Imaging (MALDI-MSI) on matching formalin-fixed paraffin-embedded (FFPE) HGSOC tissues at the time of diagnosis and following relapse with chemotherapy-resistant disease (n = 4). We identified *m*/*z* values that were differentially abundant in the matching diagnosis and relapse HGSOC tissues. These were matched to proteins using nano-liquid chromatography tandem mass spectrometry (LC-MS/MS). We identified upregulated proteins in the HGSOC relapse tissues, including COL12A1, FUBP1, PLEC, SLC4A1, and TKT. These proteins were validated by immunohistochemistry (IHC) and gene expression using online databases. IHC showed COL12A1, FUBP1, PLEC, SLC4A1, and TKT protein abundance were significantly elevated in HGSOC relapse tissues compared to matching tissues at diagnosis. *COL12A1*, *FUBP1*, *PLEC*, and *TKT* mRNA expression levels were significantly increased in HGSOC compared to normal ovary and associated with poor prognosis in HGSOC. We confirmed that higher protein abundance of both COL12A1 and PLEC correlated with reduced progression-free survival in HGSOC patients. Furthermore, both COL12A1 and PLEC mRNA and protein levels were significantly associated with chemotherapy resistance. In summary, using MALDI-MSI, we have identified proteins, including COL12A1 and PLEC, associated with chemotherapy resistance to be further evaluated as HGSOC biomarkers and/or therapeutic targets.

## 1. Introduction

Ovarian cancer is the most lethal gynecological cancer. An estimated 12,740 deaths from ovarian cancer occurred in the United States in 2024, with a 5-year survival rate of 51% [1]. High-grade serous ovarian cancer (HGSOC) is the most diagnosed subtype, accounting for 75% of the cases, and most patients are diagnosed at an advanced stage when the disease has already spread beyond the ovaries. HGSOC patients have a good response to first-line platinum-based chemotherapy treatment; however, patients develop recurrence and chemotherapy resistance [2]. The development of HGSOC platinum resistance mechanisms includes alteration of drug influx and efflux pathways, DNA repair, epigenetic alterations, and others [3]. Recent advances include treatment with poly (ADP-ribose) polymerase (PARP) and angiogenesis inhibitors. However, resistance to these therapies also develops [4]. Limited effective treatment options are available for the chemoresistant disease, which is the major cause of death for HGSOC patients. The identification of novel treatment strategies for chemoresistant ovarian cancer is urgently needed.

Matrix-Assisted Laser Desorption/Ionization–Mass Spectrometry Imaging (MALDI-MSI) is a label-free method for mapping the spatial distribution of proteins and peptides in intact tissue sections [5]. MALDI-MSI, a high-throughput approach that maintains tissue morphology, allows the detection of multiple molecules in a single tissue section and specific molecular profiling of a particular tissue [5]. One of the first studies using MALDI-MSI on HGSOC FFPE tissues combined with LC-MS/MS identified up to 297 proteins per sample [6]. Aoki et al. demonstrated comparable MSI findings between frozen and FFPE ovarian cancer tissues [7]. MALDI-MSI has been used for the identification of novel biomarkers for ovarian cancer diagnosis [8], classification of ovarian cancer subtypes [9,10,11,12], and predicting ovarian cancer recurrence [13]. Moreover, MALDI-MSI was used to investigate lipids’ spatial distribution in mouse models of HGSOC [14] and spatial visualization of N-glycans in HGSOC FFPE tissues [15,16].

To the best of our knowledge, this is the first study that utilizes MALDI-MSI and LC-MS/MS in matching diagnostic and relapse HGSOC FFPE patient tissues for the identification of novel proteins associated with chemotherapy resistance. Proteins including collagen alpha 1(XII) (COL12A1), far upstream element binding protein 1 (FUBP1), plectin (PLEC), band 3 anion transport protein (SLC4A1), and transketolase (TKT) were found to be increase in relapse HGSOC tissues compared to matching tissues at diagnosis. We validated the expression of proteins of interest (COL12A1, FUBP1, PLEC, SLC4A1, and TKT) using immunohistochemistry (IHC) and further characterized their expression in online ovarian cancer databases. We also investigated the relationship between expression with ovarian cancer patient outcome and chemotherapy resistance. Using MALDI-MSI, we have identified proteins, including COL12A1 and PLEC, associated with chemotherapy resistance to be further evaluated as HGSOC biomarkers and/or therapeutic targets.

## 2. Results

### 2.1. Identification of Proteins of Interest in Matching HGSOC Tissues at Diagnosis and Relapse Using MALDI-MSI

MALDI-MSI was performed on the matching HGSOC FFPE tissues at diagnosis and following relapse (n = 4). Peak groups representing the imaged peptides across the analyzed tissue sections were detected as described previously [17]. To identify peptides of interest for MALDI-MSI, nano-LC-MS/MS was performed on the matching HGSOC FFPE tissues (n = 4). We identified up to 108 *m*/*z* peaks (range 864.64 to 2935.625) that were differentially increased in the HGSOC diagnosis tissues (Supplementary Table S1) and up to 94 *m*/*z* peaks (range 844.541 to 2935.665) that were differentially increased in the HGSOC relapse tissues (Supplementary Table S2). MALDI-MSI *m*/*z* peaks were aligned to experimental *m*/*z* values of the sequenced peptides from nano-LC-MS/MS data. Matching was performed for peptides that fell within ± 0.25 Da *m*/*z* range of each targeted MALDI-MSI peak using LC-MS/MS data from pooled diagnosis (Supplementary Table S3) and relapse samples (Supplementary Table S4). The MALDI-MSI and LC-MS/MS analysis found collagen alpha-1(XII) chain (COL12A1) and plectin (PLEC) were increased in all four relapse tissues while far upstream element binding protein 1 (FUBP1), band 3 anion transport protein (SLC4A1), and transketolase (TKT) were increased in at least three of the four relapse tissues compared to the tissues at diagnosis (Supplementary Table S5). Collagen alpha-3(VI) chain (COL6A3) was found to be increased in all diagnosis tissues while T-complex protein 1 subunit theta (CCT8), clathrin heavy chain 1 (CLTC), fibrinogen alpha chain (FGA), isocitrate dehydrogenase [NADP], mitochondrial (IDH2), keratin, type II cytoskeletal 4 (KRT4), and myosin-9 (MYH9) were increased in three of the four diagnosis tissues (Supplementary Table S6).

**Figure 1:**
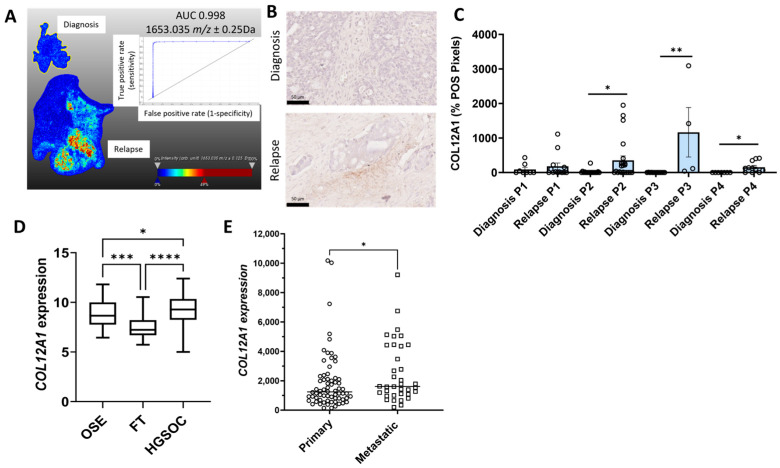
Characterization of COL12A1 expression in HGSOC. (**A**). MALDI-MSI data was acquired to generate the ion intensity maps using SCiLS lab software. Representative MALDI-MSI image for 1653.035 *m*/*z* ± 0.25 Da, tryptic peptide identified as COL12A1 was increased in HGSOC relapse tissue compared to the matching tissue at diagnosis (patient 2). Ion intensity scale ranges from blue (lowest) to red (highest). Corresponding ROC curve with AUC of 0.998 indicating the sensitivity and specificity. (**B**). Representative images of COL12A1 immunostaining in HGSOC matching tissues at diagnosis (top) and relapse with chemotherapy-resistant disease (bottom), scale bar = 50 µm. (**C**). Quantitation of COL12A1 immunostaining using QuPath in matching HGSOC tissues at diagnosis and relapse (n = 4). COL12A1 determined by the % positive pixels, * *p* < 0.05, ** *p* < 0.001 Paired T test. (**D**). *COL12A1* expression in normal tissues, including ovarian surface epithelium (OSE, n = 66), fallopian tube (FT, n = 40), and HGSOC (n = 806) from the GENT2 database. Data presented as median with min and max values, * *p* < 0.01, *** *p* < 0.001, **** *p* < 0.0001, Kruskal–Wallis with Dunn’s Multiple Comparison Test. (**E**). Increased *COL12A1* expression in metastatic HGSOC tumors (n = 36) compared to the primary HGSOC (n = 68) tumors from the GSE2109 database. Data presented as median values, * *p* < 0.05, Mann–Whitney U test.

**Figure 2:**
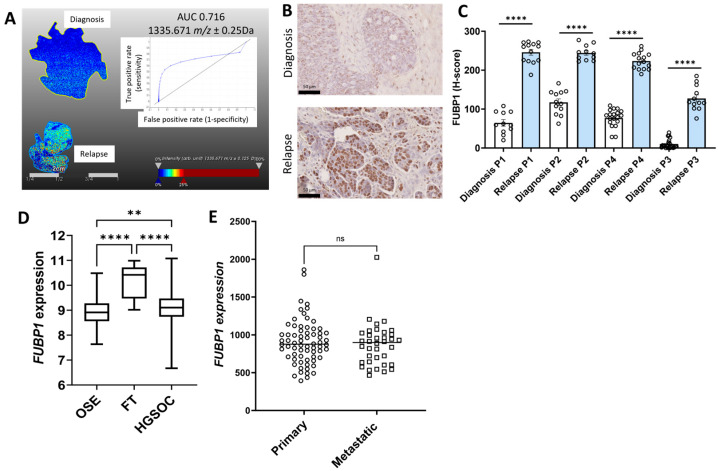
Characterization of FUBP1 expression in HGSOC. (**A**). MALDI-MSI data was acquired to generate the ion intensity maps using SCiLS lab software. Representative MALDI-MSI image for 1335.671 *m*/*z* ± 0.25 Da, tryptic peptide identified as FUBP1 was increased in HGSOC relapse tissue compared to the matching tissue at diagnosis (patient 4). Ion intensity scale ranges from blue (lowest) to red (highest). Corresponding ROC curve with AUC of 0.716 indicating the sensitivity and specificity. (**B**). Representative images of FUBP1 immunostaining in HGSOC matching tissues at diagnosis (top) and recurrence with chemotherapy-resistant disease (bottom), scale bar = 50 µm. (**C**). Quantitation of FUBP1 immunostaining in matching HGSOC tissues at diagnosis and relapse (n = 4) measured using QuPath and presented as H-score (0–300), **** *p* < 0.0001, Paired T test. (**D**). *FUBP1* expression in normal tissues, including ovarian surface epithelium (OSE, n = 66), fallopian tube (FT, n = 40), and HGSOC (n = 806) from the GENT2 database. Data presented as median with min and max values, ** *p* < 0.01, **** *p* < 0.0001, Kruskal–Wallis with Dunn’s Multiple Comparison Test. (**E**). *FUBP1* expression in primary HGSOC (n = 68) compared to metastatic tumors (n = 36) from the GSE2109 database. Data presented as median values. ns = not significant, Mann–Whitney U test.

**Figure 3:**
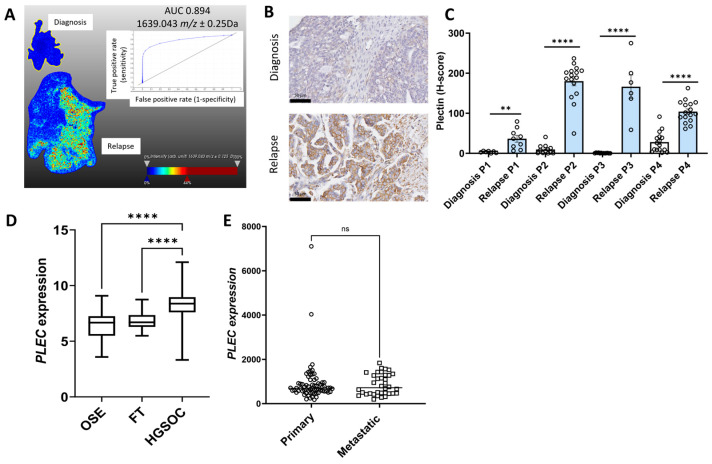
Characterization of PLEC expression in HGSOC. (**A**). MALDI-MSI data was acquired to generate the ion intensity maps using SCiLS lab software. Representative MALDI-MSI image for 1639.043 *m*/*z* ± 0.25 Da, tryptic peptide identified as PLEC was increased in HGSOC relapse tissue compared to the matching tissue at diagnosis (patient 2). Ion intensity scale ranges from blue (lowest) to red (highest). Corresponding ROC curve with AUC of 0.894 indicating the sensitivity and specificity. (**B**). Representative images of PLEC immunostaining in HGSOC matching tissue at diagnosis (top) and recurrence with chemotherapy-resistant disease (bottom), scale bar = 50 µm. (**C**). Quantitation of PLEC immunostaining in matching HGSOC tissues at diagnosis and relapse (n = 4) measured using QuPath and presented as H-score (0–300). ** *p* < 0.001, **** *p* < 0.0001, Paired T test. (**D**). *PLEC* expression in normal tissues, including ovarian surface epithelium (OSE, n = 66), fallopian tube (FT, n = 40), and HGSOC (n = 806) from the GENT2 database. Data presented as median with min and max values, **** *p* < 0.0001, Kruskal–Wallis with Dunn’s Multiple Comparison Test. (**E**). *PLEC* expression in primary HGSOC (n = 68) compared to metastatic tumors (n = 36) from the GSE2109 database. Data presented as median values. ns = not significant, Mann–Whitney U test.

**Figure 4:**
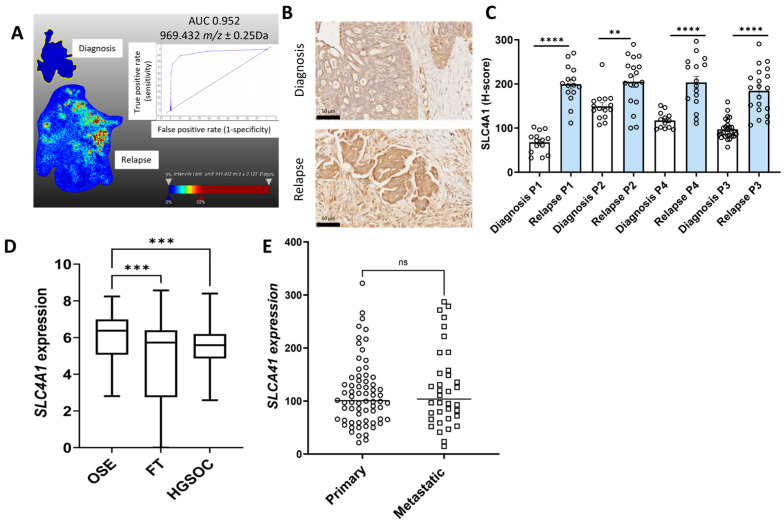
Characterization of SLC4A1 expression in HGSOC. (**A**). MALDI-MSI data was acquired to generate the ion intensity maps using SCiLS lab software. Representative MALDI-MSI image for 969.432 *m*/*z* ± 0.25 Da, tryptic peptide identified as SLC4A1 was increased in HGSOC relapse tissue compared to the matching tissue at diagnosis (patient 2). Ion intensity scale ranges from blue (lowest) to red (highest). Corresponding ROC curve with AUC of 0.9523 indicating the sensitivity and specificity. (**B**). Representative images of SLC4A1 immunostaining in HGSOC matching tissue at diagnosis (top) and recurrence with chemotherapy-resistant disease (bottom), scale bar = 50 µm. (**C**). Quantitation of SLC4A1 immunostaining in matching HGSOC tissues at diagnosis and relapse (n = 4) measured using QuPath and presented as H-score (0–300). ** *p* < 0.001, **** *p* < 0.0001, Paired T test. (**D**). *SLC4A1* expression in normal tissues, including ovarian surface epithelium (OSE, n = 66), fallopian tube (FT, n = 40), and HGSOC (n = 806) from the GENT2 database. Data presented as median with min and max values, *** *p* < 0.001, Kruskal–Wallis with Dunn’s Multiple Comparison Test. (**E**). *SLC4A1* expression in primary HGSOC (n = 68) compared to metastatic tumors (n = 36) from the GSE2109 database. Data presented as median values. ns = not significant, Mann–Whitney U test.

**Figure 5:**
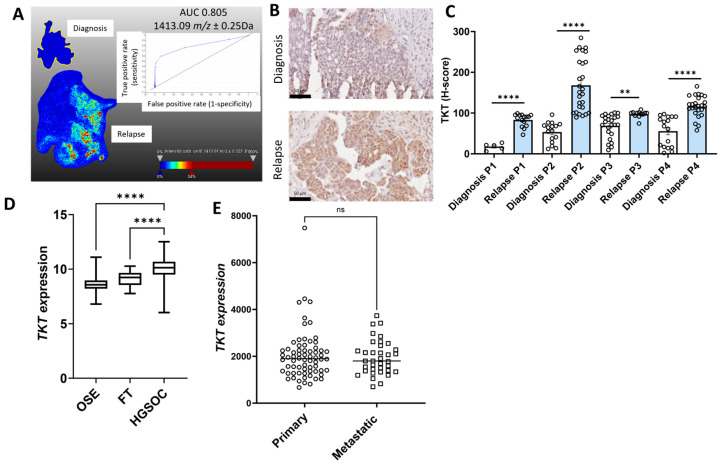
Characterization of TKT expression in HGSOC. (**A**). MALDI-MSI data was acquired to generate the ion intensity maps using SCiLS lab software. Representative MALDI-MSI image for 1413.09 *m*/*z* ± 0.25 Da, tryptic peptide identified as TKT was increased in HGSOC relapse tissue compared to the matching tissue at diagnosis (patient 2). Ion intensity scale ranges from blue (lowest) to red (highest). Corresponding ROC curve with AUC of 0.805 indicating the sensitivity and specificity. (**B**). Representative images of TKT immunostaining in HGSOC matching tissue at diagnosis (top) and recurrence with chemotherapy-resistant disease (bottom), scale bar = 50 µm. (**C**). Quantitation of TKT immunostaining in matching HGSOC tissues at diagnosis and relapse (n = 4) using QuPath and presented as H-score (0–300). ** *p* < 0.001, **** *p* < 0.0001, Paired T test. (**D**). *TKT* expression in normal tissues, including ovarian surface epithelium (OSE, n = 66), fallopian tube (FT, n = 40), and HGSOC (n = 806) from the GENT2 database. Data presented as median with min and max values, **** *p* < 0.0001, Kruskal–Wallis with Dunn’s Multiple Comparison Test. (**E**). *TKT* expression in primary HGSOC (n = 68) compared to metastatic tumors (n = 36) from the GSE2109 database. Data presented as median values. ns = not significant, Mann–Whitney U test.

Representative MALDI-MSI ion intensity images with *m*/*z* values and receiver operating curves (ROCs) that were significantly increased in relapse HGSOC tissues compared to the matching tissues at diagnosis are shown for COL12A1 (Figure 1A), FUBP1 (Figure 2A), PLEC (Figure 3A), SLC4A1 (Figure 4A), and TKT (Figure 5A). The ion intensity maps represent the abundance of *m*/*z* value, which ranges from low (blue) to high (red). The area under the curve (AUC) for the ROC curve shows the discriminatory power of the *m*/*z* value, ranging from 0 to 1. The closer the AUC value to 1 indicates a good discrimination between the two groups. *m*/*z* 1653.035 with an AUC value of 0.998 was more abundant in patient 2 relapse tissue compared to the matched diagnosis tissue with COL12A1 peptide GPGDLEAPSNLVISER (Figure 1A). *m*/*z* 1530.705 identified in patients 2, 3, and 4 matched to a COL12A1 peptide (VSWDPSPSPVLGYK), and *m*/*z* 844.541 identified only in patient 1 also matched to a COL12A1 peptide (AADAKELK) (Supplementary Table S5). *m*/*z* 1335.671 (AUC = 0.716) with a higher abundance in patient 3 and 4 relapse tissues compared to the diagnosis tissue matched with FUBP1 peptide IGGNEGIDVPIPR (Figure 2A). *m*/*z* 930.462 identified in patients 2 and 4 also matched to a FUBP1 peptide (FAVGIVIGR) (Supplementary Table S5).

Similarly other *m*/*z* values more abundant in tissues at relapse (patient 2 and 4) compared to diagnosis included *m*/*z* 1639.043 (peptide VPLDEALQRGTVDAR) (AUC = 0.894) (Figure 3A), *m*/*z* values 1298.701 (peptide VTLVQTLEIQR, patient 1), *m*/*z* 1298.815 (peptide VTLVQTLEIQR, patient 1), *m*/*z* 1459.938 (peptide SLESLHSFVAAATK, patient 2 and 4), *m*/*z* 1530.705 (peptide VLALPEPSPAAPTLR, patient 2, 3 and 4), *m*/*z* 1565.93 (peptide APVPASELLASGVLSR, patient 2), and *m*/*z* 1612.083 (EAEGQLQKLQEALR, patient 2) all matched with PLEC peptides (Supplementary Table S5). *m*/*z* 969.432 (AUC = 0.952), which matched to a SLC4A1 peptide VLLPLIFR, was identified in patient 2 and patient 4 (Figure 4A, Supplementary Table S5). *m*/*z* 1489.89 identified in patients 3 and 4 also matched to a SLC4A1 peptide (ADFLEQPVLGFVR). *m*/*z* 1413.09 (AUC = 0.805) identified in patients 2 and 4 matched with a TKT peptide VLDPFTIKPLDR (Figure 5A, Supplementary Table S5). Other m/z values identified in patients 2 and 4, *m*/*z* 1413.057 and *m*/*z* 1561.816, in patients 2, 3, and 4 also matched with TKT peptides (Supplementary Table S5).

### 2.2. Validation of Protein of Interest Increased in Relapse Tissues Compared to Diagnosis Using IHC

The expression for the five proteins of interest (COL12A1, FUBP1, PLEC, SLC4A1, and TKT) increased in relapse tissues was validated using IHC. Minimal COL12A1 protein was present in HGSOC tissues at diagnosis, but COL12A1 protein levels (% positive pixels) were significantly increased in the cancer-associated stroma in patients 2, 3, and 4 but not in patient 1 relapse tissues compared to tissues at diagnosis (Figure 1B,C). FUBP1 predominantly expressed in cancer cell nuclei was significantly increased in all four matching HGSOC relapse tissues compared to tissues at diagnosis (Figure 2B,C). PLEC expressed in the cytoplasm and membrane of cancer cells was significantly increased in all four matching relapse tissues compared to tissues at diagnosis (Figure 3B,C). SLC4A1 predominately expressed in the cytoplasm of cancer cells was significantly increased in all four relapse tissues compared to tissues at diagnosis (Figure 4B,C). TKT expression in cancer cell cytoplasm and nuclei was significantly increased in all four relapse tissues compared to tissues at diagnosis (Figure 5B,C).

### 2.3. Characterization of the Expression Proteins of Interest in Online Ovarian Cancer Databases

mRNA expression levels of *COL12A1*, *FUBP1*, *PLEC*, *SLC4A1*, and *TKT* were investigated further using the online GENT2 database [18]. Expression of *COL12A1* (Figure 1D), *PLEC* (Figure 3D), and *TKT* (Figure 5D) were significantly increased in HGSOC compared to ovarian surface epithelium (OSE) and fallopian tube (FT). *FUBP1* expression was increased in HGSOC compared to OSE but decreased compared to FT (Figure 2D). *SLC4A1* expression was decreased in HGSOC compared to OSE and not different compared to FT (Figure 4D).

We additionally compared the expression of these proteins of interest in primary and metastatic HGSOC tumors using the GSE2109 database. *COL12A1* expression was significantly increased in metastatic compared to primary HGSOC tumors (Figure 1E). No difference was observed for *FUBP1* (Figure 2E), *PLEC* (Figure 3E), *SLC4A1* (Figure 4E), and *TKT* (Figure 5E) expression between the primary and metastatic HGSOC tumors.

### 2.4. Relationship Between Proteins of Interest with HGSOC Patient Outcome

Increased expression of *CO12A1*, *FUBP1*, and *TKT* but not *PLEC* or *SLC4A1* were significantly associated with reduced progression-free survival (PFS) in HGSOC patients using the Kaplan–Meier online plotter database (Table 1). Increased expression of *CO12A1* and *TKT* was also associated with reduced overall survival (OS), and increased expression of *PLEC* was associated with reduced post-progression survival (PPS) (Table 1).

**Figure 6:**
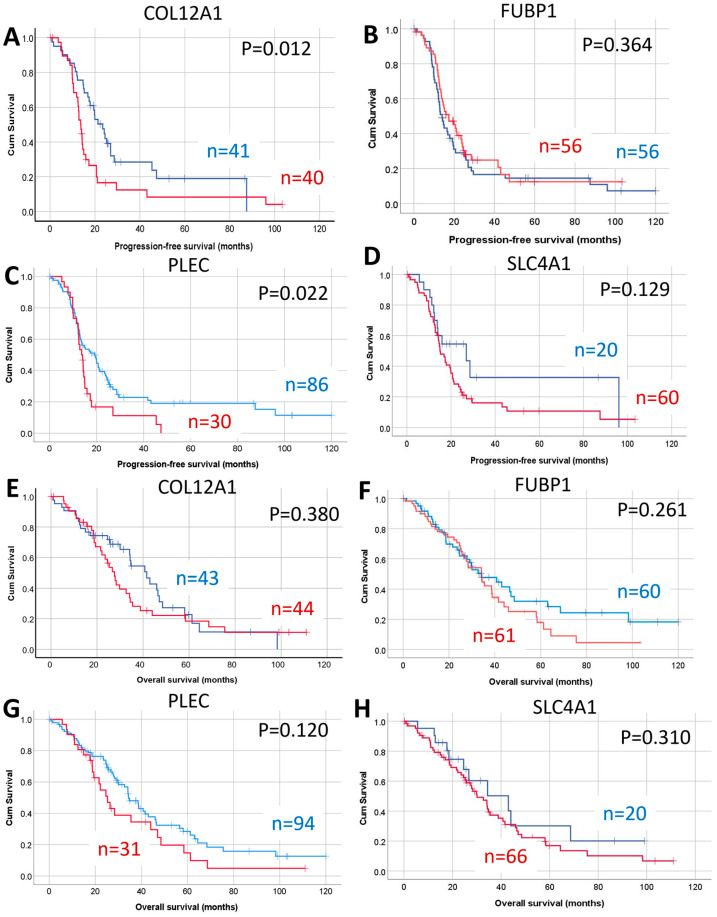
Relationship between COL12A1, FUBP1, PLEC, and SLC4A1 protein levels and HGSOC patient outcome. Relationship between the protein levels for (**A**) COL12A1 cut-off pos vs. neg, (**B**) FUBP1 cut-off H-score median max (≤107.6 vs. >107.6), (**C**) PLEC H-score max Q1–Q3 vs. Q4 (≤185 vs. >185) and (**D**) SLC4A1 H-score Q1 vs. Q2–Q4 (≤17.1 and >17.1) with progression-free survival (PFS) in HSGOC patient cohort. Relationship between the protein levels for (**E**) COL12A1 cut-off pos vs. neg, (**F**) FUBP1 cut-off H-score median max ≤ 107.6 vs. >107.6, (**G**) PLEC cut-off H-score max Q1–Q3 vs. Q4 (≤185 vs. >185), and (**H**) SLC4A1 H-score Q1 vs. Q2–Q4 (≤17.1 and >17.1) with overall survival (OS) in HSGOC patient cohort.

We further assessed protein levels of COL12A1, FUBP1, PLEC, and SLC4A1 in an independent HGSOC TMA cohort using IHC. TKT protein levels were not assessed in this study as we have previously reported that TKT protein levels were increased in metastatic HGSOC and associated with reduced OS [19]. COL12A1, FUBP1, PLEC, and SLC4A1 proteins were variably expressed in the HGSOC tumors. Examples of HGSOC tissues with low and high immunostaining for COL12A1, FUBP1, PLEC, and SLC4A1 are shown in Supplementary Figure S1. The presence of positive COL12A1 immunostaining in the cancer-associated stroma was associated with reduced PFS (Figure 6A, *p* = 0.012), but no significant relationship was observed between COL12A1 positivity with OS (Figure 6E). There was no significant relationship between FUBP1 protein levels with either PFS (Figure 6B) or OS (Figure 6F) in HGSOC. Increased levels of PLEC were also associated with reduced PFS (Figure 6C, *p* = 0.022) but not with OS (Figure 6G) in the HGSOC patient cohort. SLC4A1 protein levels in HGSOC were not associated with either PFS (Figure 6D) or OS (Figure 6H).

### 2.5. COL12A1, PLEC, and SLC4A1 Expressions Associated with Chemotherapy Resistance

**Figure 7:**
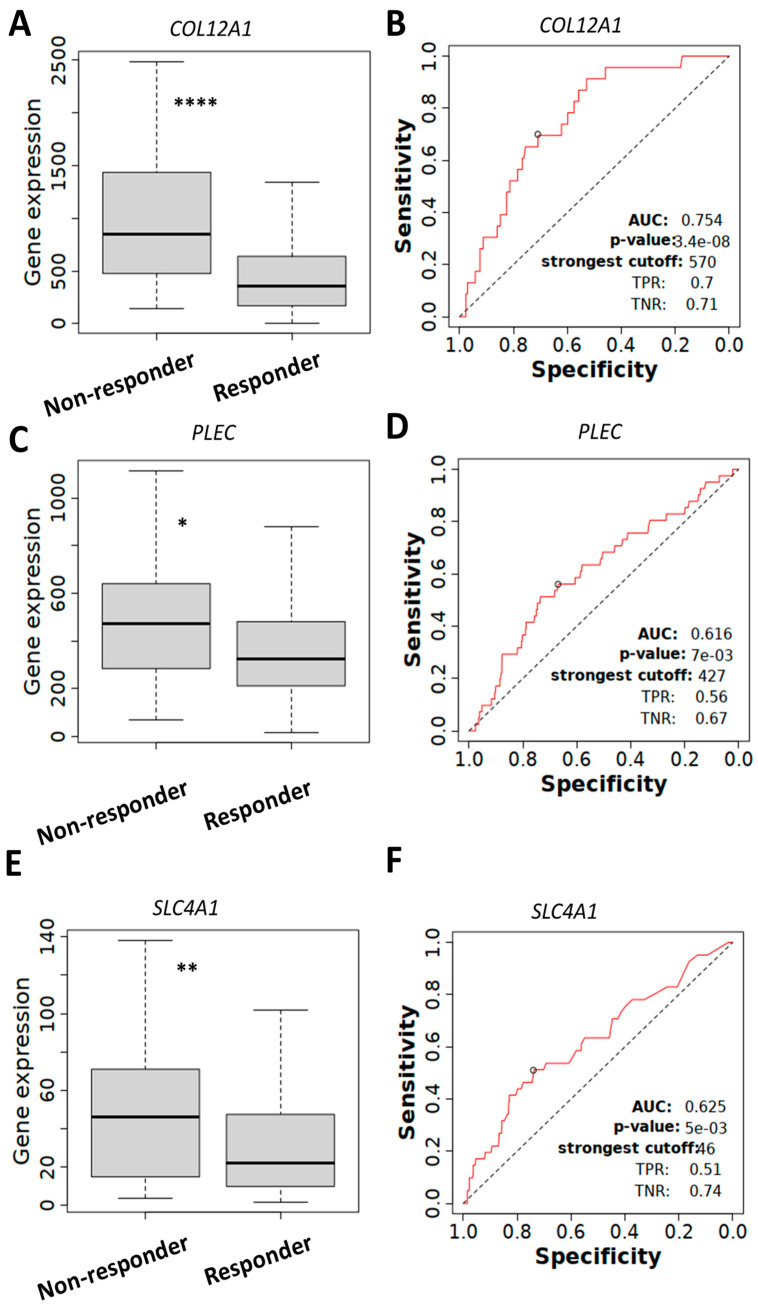
Increased *COL12A1*, *PLEC*, and *SLC4A1* expression in serous ovarian cancer (grade 3) non-responders patients treated with platinum and taxane chemotherapy. (**A**). Increased *COL12A1* (231879_at) expression in serous ovarian cancer non-responders (n = 23) compared to responders (n = 172), Mann–Whitney U test, **** *p* < 0.0001. (**B**). Relationship between *COL12A1* expression and therapy response. Area under the curve (AUC) = 0.754, *p* = 3.4 × 10^−8^. (**C**). Increased *PLEC* (201373_at) expression in serous ovarian cancer non-responders (n = 41) compared to responders (n = 544), Mann–Whitney U test * *p* < 0.05. (**D**). Relationship between *PLEC* expression and therapy response, AUC = 0.616, *p* = 0.007. (**E**). Increased *SLC4A1* (205592_at) expression in serous ovarian cancer non-responders (n = 169) compared to responders (n = 241), Mann–Whitney U test, ** *p* < 0.001. (**F**). Relationship between *SLC4A1* expression and therapy response, AUC = 0.625, *p* = 0.005. Data obtained from the ROC plotter database.

Next, we evaluated whether mRNA expression levels of the proteins of interest were associated with chemotherapy resistance using the ROC plotter database. *COL12A1* (Figure 7A), *PLEC* (Figure 7C), and *SLC4A1* (Figure 7E) expression levels were significantly increased in serous ovarian tumors (grade 3) that did not respond to platinum and taxane chemotherapy compared to responders after 6 months of treatment. The ROC curve analysis confirmed a significant relationship between the expression of *COL12A1* (Figure 7B, AUC = 0.754), *PLEC* (Figure 7D, AUC = 0.616), and *SLC4A1* (Figure 7F, AUC = 0.625) with chemotherapy response. 

**Figure 8:**
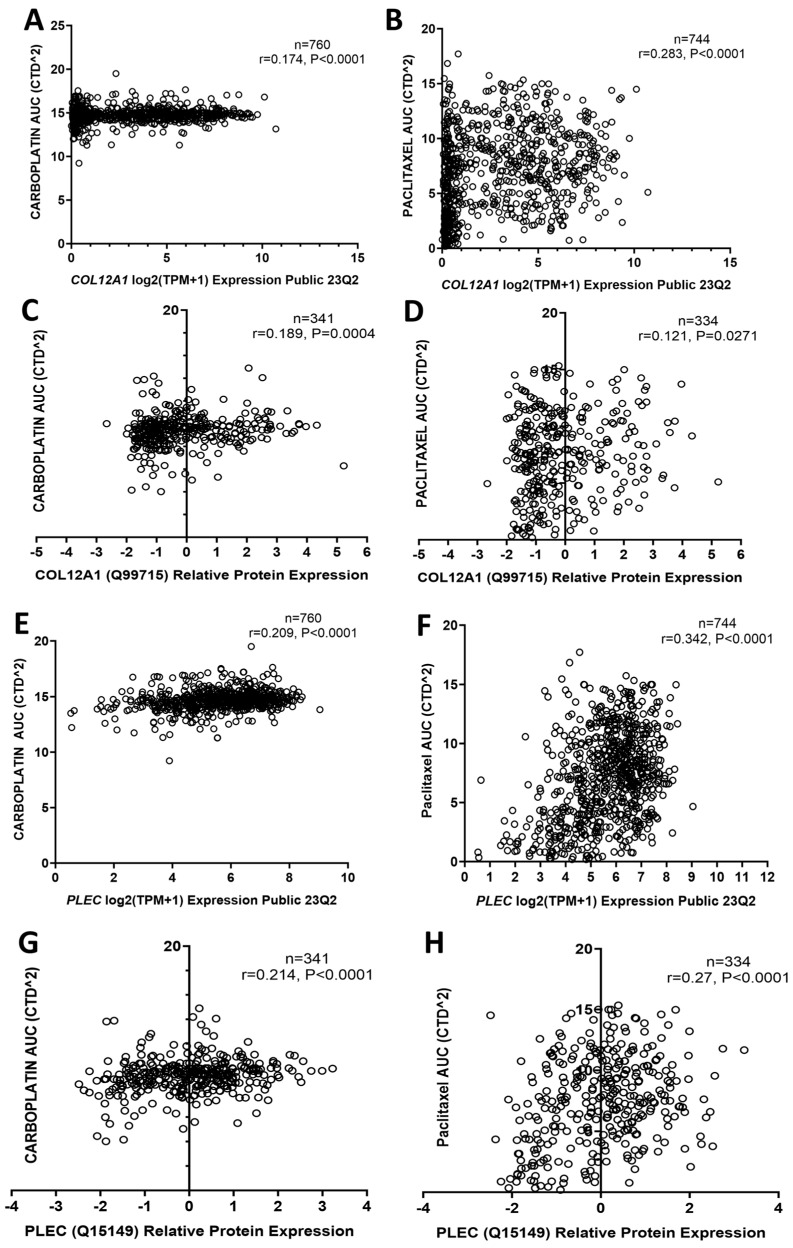
COL12A1 and PLEC expression is associated with carboplatin and paclitaxel resistance in cancer cell lines. (**A**). Correlation between *COL12A1* mRNA expression and carboplatin AUC in cancer cell lines (n = 760). (**B**). Correlation between *COL12A1* mRNA expression and paclitaxel AUC in cancer cell lines (n = 744). (**C**). Correlation between COL12A1 protein levels and carboplatin AUC in cancer cell lines (n = 341). (**D**). Correlation between COL12A1 protein levels and paclitaxel AUC in cancer cell lines (n = 334). (**E**). Correlation between *PLEC* mRNA expression and carboplatin AUC in cancer cell lines (n = 760). (**F**). Correlation between *PLEC* mRNA expression and paclitaxel AUC in cancer cell lines (n = 744). (**G**). Correlation between PLEC protein levels and carboplatin AUC in cancer cell lines (n = 341). (**H**). Correlation between PLEC protein levels and paclitaxel AUC in cancer cell lines (n = 334). Data obtained from the DepMap database.

We assessed data from the DepMap portal database to investigate the relationship between gene and protein expression with response to both carboplatin and paclitaxel treatment. COL12A1 mRNA and protein expression were positively associated with carboplatin AUC (Figure 8A,C) and paclitaxel AUC (Figure 8B,D) in all cancer cell lines, suggesting a relationship with both carboplatin and paclitaxel resistance. Similarly, PLEC mRNA and protein expression were also positively associated with carboplatin AUC (Figure 8E,G) and paclitaxel AUC (Figure 8F,H) in all cancer cell lines Data from the GSE45553 database showed the expression of *COL12A1*, *PLEC*, and *TKT* in cisplatin-resistant OVCAR-8 spheroids were significantly increased compared to OVCAR8 control spheroids, also supporting a role for COL12A1, PLEC, and TKT in chemotherapy resistance (Supplementary Figure S3).

## 3. Discussion

We showed in this study that MALDI-MSI can be used to identify proteins associated with chemotherapy resistance in HGSOC FFPE tissues. *m*/*z* values that were differentially abundant in matching HGSOC tissues at diagnosis and relapse were matched to protein peptides using nano-LC-MS/MS. We identified five proteins, including COL12A1, FUBP1, PLEC, SLC4A1, and TKT, that were upregulated in at least three out of four HGSOC relapse tissues. These proteins were validated in HGSOC patient tissues using IHC and in online ovarian cancer databases analyzing mRNA data. Validation studies using IHC showed COL12A1, FUBP1, SLC4A1, and TKT levels were significantly increased in the HGSOC relapse tissues compared to matching tissues at diagnosis. *COL12A1*, *FUBP1*, *PLEC*, and *TKT* expression in HGSOC were significantly increased compared to normal ovaries and associated with poor prognosis. Our analyses using an ROC plotter database demonstrated increased *COL12A1*, *PLEC*, and *SLC4A1* expression associated with resistance to platinum and taxane chemotherapy. COL12A1 and PLEC expression correlated with resistance to carboplatin and paclitaxel treatment. This study has identified proteins associated with chemotherapy resistance in HGSOC that can be further evaluated as biomarkers and/or therapeutic targets.

Our MALDI-MSI analyses demonstrated increased COL12A1 levels in relapse HGSOC tissues compared to tissues at diagnosis, which was validated by IHC. Analyses of the ROC database also showed increased *COL12A1* expression in serous ovarian cancer patients who did not respond to platinum and taxane chemotherapy. Moreover, COL12A1 mRNA expression and protein levels in cancer cell lines associated with carboplatin and paclitaxel resistance and increased *COL12A1* expression in OVCAR-8 spheroids cisplatin-resistant cells also support a relationship with chemotherapy resistance. Increased *COL12A1* expression has also been reported in ovarian cancer A2780 cisplatin-resistant (23.9-fold change increased) and A2780 doxorubicin-resistant cell lines (16.4-fold change increased) compared to parental A2780 cells [20]. Yan et al. showed that increased *COL12A1* expression was associated with resistance to PD-L1 inhibitors, durvalumab, and poor patient outcomes in breast cancer [21]. However, using proteomics, COL12A1 was identified as one of the down-regulated proteins in cisplatin-resistant ovarian cancer cell lines [22]. Together these studies suggest a relationship between COL12A1 and ovarian cancer chemotherapy resistance but warrants further investigation.

Using online databases, increased *COL12A1* mRNA expression in HGSOC compared to OSE and FT and increased *COL12A1* mRNA expression in metastatic compared to primary HGSOC tissues was observed. In contrast, Chudasama et al. reported no difference in *COL12A1* expression between ovarian cancer and healthy control tissues using qRT-PCR [23]. In agreement with our findings, another study found that high *COL12A1* expression in ovarian cancer was associated with reduced OS [23]. Serum COL12A1 levels were elevated in solid tumor patients compared to healthy controls [24]. Proteomics and single-cell transcriptomics analyses of genetically engineered mouse models identified increased levels of COL12A1 in breast cancers compared to normal mammary tissues [25]. COL12A1 was found to play an important role in promoting breast cancer metastasis [25]. COL12A1 was also reported to promote gastric cancer metastasis via the MAPK pathway [26]. Future studies are warranted to further investigate the role of COL12A1 and its potential as a therapeutic target in HGSOC.

MALDI-MSI and IHC analysis demonstrated increased FUBP1 expression in the HGSOC relapse tissue compared to tissues at diagnosis. Our online database analyses showed increased *FUBP1* expression in HGSOC compared to the OSE, and high *FUBP1* expression was associated with poor HGSOC outcome. Similarly, analyses using online databases by others also showed increased *FUBP1* expression in ovarian cancer compared to normal ovarian tissues [27,28]. In addition, previous studies have shown increased FUBP1 expression in ovarian cancer tissues compared to normal tissues using IHC [28,29,30]. FUBP1 was overexpressed in various tumor types and plays a role in cell proliferation, cell migration and invasion, and cell apoptosis [31]. From our online database analyses, *FUBP1* expression was not associated with chemoresistance. However, previous studies reported that knockdown of FUBP1 in SKOV-3 ovarian cancer cells reduced tumor burden in a nude mouse model [27]. Increased FUBP1 expression in SKOV-3 cells was associated with cisplatin resistance [27]. Zhang et al. also reported that silencing FUBP1 in SKOV-3 ovarian cancer cells enhanced sensitivity to carboplatin [30]. Further studies are warranted to investigate the role of FUBP1 in ovarian cancer.

Plectin has been shown to have both tumor-suppressive and tumor-promoting roles [32,33]. Our findings demonstrating increased *PLEC* expression in HGSOC compared to OSE and FT and a relationship between high expression and poor HGSOC patient outcome support a tumor-promoting role. Our online database analyses showed increased *PLEC* expression in serous ovarian cancer patients who did not respond to platinum and taxane chemotherapy, a positive relationship with carboplatin and paclitaxel resistance in cancer cell lines, and increased *PLEC* expression in OVCAR-8 cisplatin-resistant spheroids, suggesting a relationship with chemotherapy resistance. Increased plectin expression using IHC was also observed in recurrent HEY ovarian cancer cell line-derived mouse xenografts tissues compared to untreated control and paclitaxel-treated xenografts [34]. However, plectin was down-regulated in chemoresistant ascites-derived ovarian cancer cells by proteomics analyses [35]. Increased plectin protein levels were observed in type I (low-grade tumors) ovarian cancer tissues compared to type II (high-grade serous ovarian tumors) [34,36]. Monoclonal antibodies targeting cancer-specific plectin reduced ovarian cancer cell migration increased chemosensitivity to cisplatin, and reduced tumor growth in mouse models [37]. Moreover, plectin-targeted liposomes enhanced the therapeutic efficacy of PARP inhibitors in ovarian cancer treatment [38]. Further studies are warranted to address the role of plectin in HGSOC progression, chemoresistance, and its potential as a therapeutic target.

Limited studies have investigated SLC4A1 expression and its functional role in ovarian cancer. We observed decreased *SLC4A1* expression in HGSOC compared to normal ovarian tissue and no difference compared to FT. RNA sequencing data showed decreased *SLC4A1* expression in cancers of the lung, colorectal, and breast [39]. However, increased SLC4A1 cytoplasm expression was observed in gastric and colorectal cancers [40]. The online ROC plotter database demonstrated increased *SLC4A1* expression in serous ovarian cancer patients who did not respond to platinum and taxane chemotherapy. Bioinformatics analyses of ovarian cancer publicly available gene expression databases and machine learning identified *SCL4A1* as one of the genes that were upregulated in ovarian cancer patients with platinum resistance [41]. Further studies are warranted to investigate the role of SLC4A1 in ovarian cancer.

In this study, increased TKT expression was observed in relapse HGSOC tissues compared to matching tissues at diagnosis using both MALDI-MSI and IHC. TKT plays an important role in tumor progression and metastasis in various tumor types [42]. *TKT* expression was increased in HGSOC compared to OSE and FT, and high *TKT* expression was associated with reduced HGSOC patient outcomes. Previous studies have also reported high TKT expression associated with poor ovarian cancer patient outcomes [19,43]. The online database analysis in this study showed no difference between *TKT* expression in primary and metastatic HGSOC tissues; however, we previously reported TKT protein levels to be upregulated in metastatic peritoneal implants and promote ovarian cancer cell proliferation [19]. Proteomics analysis identified TKT as one of the proteins to be increased in ovarian cancer ascites fluid compared to benign ascites fluid [44]. TKT was identified as a diagnostic marker for ovarian cancer [45] and associated with cisplatin resistance [46]. TKT plays a key role in the pentose phosphate pathway and has been identified in ovarian cancer exosomes [47]. Future studies are warranted to investigate the role of TKT in HGSOC progression and chemoresistance.

A limitation of our study is the small sample size for the matching HGSOC tissue at diagnosis and at relapse (n = 4 pairs). The availability of the matching HGSOC tissues is very limited, and these biological samples are very valuable clinical resources. Further studies are warranted to validate the expression of the candidate proteins (COL12A1, FUBP1, PLEC, SLC4A1, and TKT) in a larger and independent HGSOC patient cohort. Extensive functional experiments are required to confirm the association of the candidate proteins with ovarian cancer chemoresistance. Future studies, including characterization of the candidate proteins in chemoresistant HGSOC tissues and cell lines and knockdown studies to investigate the importance of candidate proteins in HGSOC chemoresistant signaling pathways, are required.

## 4. Materials and Methods

### 4.1. Patient Cohorts

FFPE samples were obtained from matching HGSOC patients at diagnosis (n = 4, 2009–2013) and after recurrence with chemotherapy-resistant disease (n = 4, 2013–2015) (Supplementary Table S7). Tissue microarrays (TMA) were assembled from FFPE HGSOC tissues from patients diagnosed between 1988 and 2010 (Supplementary Table S7). The tissue samples were collected with approval by the Royal Adelaide Hospital Human Research Ethics Committee (protocols #060903, #080102 and #140101) with informed patient consent.

### 4.2. MALDI-MSI Preparation and Acquisition

MALDI-MSI was performed on matching HGSOC tissues at diagnosis and relapse (n = 4), as previously described [6]. Briefly, 5 µm FFPE tissue sections were mounted on Indium-Tin-Oxide (ITO)-coated glass slides (Bruker Daltonics, Bremen, Germany) by heating at 60 °C for 1 h. Trypsin digestion was performed using trypsin gold (Promega, Madison, WI, USA), which was applied to the tissue sections using the ImagePrep station (Bruker Daltonics, Bremen, Germany) following Bruker’s default trypsin application method with minor modifications. Specifically, trypsin was sprayed using 38% spray power with 0% modulation for a total of 30 cycles. Each spray cycle consisted of 1.25 s of spraying followed by 45 s of drying. These settings were selected based on in-house optimization to ensure uniform enzyme coverage and consistent proteolytic digestion. After application, the tissue sections were incubated at 37 °C for 2 h to allow for effective digestion. Following digestion, peptide internal standards (Angiotensin I, [Glu1]-Fibrinopeptide B, Dynorphin A, and ACTH fragment (1–24)) were applied using the same ImagePrep system [48] and under identical settings as used for trypsin application. These standards served as internal calibrants for spectral alignment and normalization across samples. Subsequently, α-cyano-4-hydroxycinnamic acid (HCCA) was applied as the MALDI matrix using Bruker’s default HCCA method on the ImagePrep, with minor modifications as described and optimized in our previously published study [49]. The matrix application protocol was specifically refined to ensure consistent and reproducible matrix crystallization and ionization efficiency, thereby supporting high-quality and comparable MSI data across all tissue sections. Data was acquired in positive reflectron mode using an UltrafleXtreme MALDI TOF/TOF instrument (Bruker Daltonics, Bremen, Germany), controlled by flexControl (V3.0.1 Bruker Daltonics, Bremen, Germany) and flexImaging software (V4.0.1 Bruker Daltonics, Bremen, Germany). The data was acquired at the laser repetition rate of 2 kHz over the mass range of *m*/*z* 800–4000, with 100 µm spatial resolution [50]. Following data acquisition, slides were stained using hematoxylin and eosin (H&E) and scanned using a NanoZoomer (Hamamatsu, Japan). The H&E-stained images with annotations were linked with the collected MALDI-MSI spectra using the flex imaging software.

### 4.3. MALDI-MSI Data Analysis

For data pre-processing, the Snap Algorithm with a signal-to-noise ratio of 2 was used for peak detection, the TopHat method was performed for baseline subtraction, and baseline smoothing was performed using the Gauss algorithm. Peak lists from all of the ROIs were combined, and density-based clustering (DBSCAN) with an epsilon of 0.02 and a minimum density of 100 peaks was used to cluster peaks into peak groups based on their *m*/*z* values [50].

Only peak groups containing at least 10,000 peaks were considered for further analysis. The median log intensity in each of the tissues was calculated for each peak group and for each patient. Peak groups were heuristically ranked by the largest difference in median log intensity between tissue types. The raw data was also analyzed using the SCiLS lab software (SCiLS, GmbH, Bremen, Germany, 2016b), and the processing steps of baseline removal and normalization were conducted as previously described [51]. SCiLS lab software was used to generate the ion intensity maps for the peptides of interest.

### 4.4. Peptide Identification by Nanoflow Liquid Chromatography Tandem Mass Spectrometry (Nano-LC-MS/MS)

The FFPE tissue was sectioned at 8 µm thickness, water bath mounted onto polyethylene naphthalate (PEN) membrane slides (Micro-Dissect, Herborn, Germany), and deparaffinized as described above. Tissue areas known to contain cancer were scrapped into 20 µL of citric acid buffer (10 mM, pH = 6) and subjected to heat-induced antigen retrieval. Samples were buffered with NH_4_HCO_3_ (10 mM) and digested with trypsin gold (100 ng, Promega, Madison, WI, USA) overnight at 37 °C. Nano-LC-MS/MS was performed using an Ultimate 3000 RSLC system (Thermo-Fisher Scientific, Waltham, MA, USA) coupled to an Impact II™ QTOF mass spectrometer (Bruker Daltonics, Billerica, MA, USA) via an Advance CaptiveSpray source (Bruker Daltonics) [50]. Acquired spectra were subjected to peak detection, de-convolution, and re-calibration according to a lock mass using Data Analysis (Version 4.2, Bruker Daltonics). Processed spectra were then exported to Mascot generic format and submitted to Mascot (Version 2.3.02) for peptide identification. Search parameters were as follows: SwissProt Homo sapiens database, the digestion enzyme with trypsin with up to two missed cleavages, variable modification of oxidation of methionine, MS mass tolerance of 40 ppm, and MS/MS mass tolerance of 0.25 Da. In the Mascot, the peptide false discovery rate was set to <0.05.

### 4.5. Matching the MALDI-MSI Peak Groups to the Nanoflow Liquid Chromatography Tandem Mass Spectrometry (Nano-LC-MS/MS)

Matching between the two data sets was performed manually by comparing the experimental *m*/*z* values of the nano-LC-MS/MS sequenced peptides that fell between the ±0.25 *m*/*z* of each of the MALDI-MSI peak groups in both the diagnosis and relapse samples [50]. SCiLS lab v2016b with edge-preserving image denoising and automatic hotspot removal applied.

### 4.6. Ovarian Cancer Online Databases

GENT2 (http://gent2.appex.kr, accessed on 7 July 2022) analysis of GPL570 platform (HG-U133) microarray data for ovarian cancer patients (n = 1626 patients, n = 35 genomic spatial events datasets) was assessed [18,52]. Individual samples were reviewed and specified as normal ovarian surface epithelium (OSE, n = 66), fallopian tube (FT, n = 40), and HGSOC (n = 806). GEO2R (https://www.ncbi.nlm.nih.gov/geo/geo2r/, accessed on 7 July 2022) were used to obtain data for primary HGSOC (n = 68) and metastatic HGSOC (n = 36) from the GSE2109 dataset. Moreover, the GSE45553 dataset was used for the mRNA expression in OVCAR-8 spheroids (n = 4) and OVCAR-8 cisplatin-resistant spheroids (n = 4).

The Dependency Map (DepMap) portal (https://depmap.org/portal/, accessed on 5 June 2023 was used to access The Cancer Cell Line Encyclopedia (CCLE, Broad Institute), which comprises information on gene and protein expression and drug responses in cancer cell lines. The gene expression data (Expression Public 23Q2) and protein data (Proteomics), along with the drug sensitivity AUC (Cancer Target Discovery and Development (CTD^2)) values for carboplatin and paclitaxel response were obtained.

ROC plotter (http://rocplot.org/ovarian/index, accessed on 12 December 2023) provides data on gene expression from microarray analysis and treatment outcomes for individuals with cancer [53]. We investigated gene expression in grade 3 serous ovarian cancer patients treated with both platinum and taxane chemotherapy. Our analysis compared gene expression levels between patients who experienced a relapse within six months after completing treatment (non-responders) and those who did not experience a relapse during the period (responders).

*COL12A1*, *FUBP1*, *PLEC*, *SLC4A1*, and *TKT* mRNA expression in public datasets were analyzed by Kaplan–Meier online plotter and used to calculate the hazard ratio, 95% CI, log-rank *p*-value and Kaplan–Meier survival curves [54]. In the Kaplan–Meier online plotter, PPS is calculated from the time of first progression to the time of death, PFS survival is calculated from the date of diagnosis to first progression, and OS is calculated from the date of diagnosis to the date of death. Probe details are *COL12A1* (225664_at, 231766_s_at, 231879_at, 234951_s_at), *FUBP1* (203091_at, 212847_at, 214093_s_at, 214094_at), *PLEC* (201373_at, 216971_s_at), *SLC4A1* (1552713_a_at, 205592_at), and *TKT* (205168_at, 208699_x_at, 208700_s_at, 228205_at). For genes with multiple probes, the mean expression was calculated, and the best cut-off setting was used to split the patients into groups with high and low expression. This analysis included ovarian cancer patients with serous subtype and grade (2 and 3).

### 4.7. IHC

IHC was performed with antibodies to COL12A1 (1/75, Ab121304, Abcam, Cambridge, UK), FUBP1 (1/1000, PA5-82235, Invitrogen, Waltham, MA, USA), PLEC (1/600, Ab32528, Abcam), SLC4A1 (1/100, 28131-1-AP, Proteintech, Rosemont, IL, USA), and TKT (1/200, H-50, sc-67120, Santa Cruz Biotechnology, Dallas, TX, USA). Slides were subsequently incubated with biotinylated goat anti-rabbit (1/400, Dako, Mulgrave, VIC, Australia) and streptavidin-HRP (1/500, Dako, Australia) each for 1 h at room temperature. Peroxidase activity was detected using the substrate diaminobenzidine (DAB)/H_2_O_2,_ as described previously [55]. Positive controls for the immunostaining included human fallopian tube (COL12A1), mouse fallopian tube (FUBP1), human placenta (PLEC), mouse kidney (SLC4A1) and HGSOC tissue that was previously positive for TKT expression (Supplementary Figure S2). Negative controls included no primary antibody or rabbit IgG isotype control at the same concentration of the primary antibody (Thermofisher Scientific, USA). Slides were digitally scanned using the NanoZoomer Digital Pathology System (Hamamatsu Photonics, Shizuoka, Japan) and viewed by NDP view imaging software (v2.3, Hamamatsu Photonics).

### 4.8. IHC Assessment

For the quantification of IHC of the matching HGSOC tissue at diagnosis and relapse and HGSOC TMA, QuPath software was used (Version 0.2.3) [56]. FUBP1, PLEC, SLC4A1, and TKT immunostaining were measured as an H-score (0–300), which represents the intensity of positive staining and percentage of positive cells in epithelial cells (5–6 areas per tissue). COL12A1 immunostaining in the cancer-associated stroma was measured using % positive pixels.

### 4.9. Statistical Analysis

Kaplan–Meier analyses were performed to assess the relationship of COL12A1, FUBP1, PLEC, and SLC4A1 protein expression in the HGSOC TMA cohort with PFS and OS (SPSS software, version 21.0, SPSS Inc., Chicago, IL, USA). GraphPad Prism (Version 9.0.0, GraphPad Software Inc., La Jolla, CA, USA) was used for the following statistical analyses, including Mann–Whitney U test, Kruskal–Wallis with Dunn’s Multiple Comparison Test, and Paired t test. Statistical significance was accepted at *p* < 0.05.

## 5. Conclusions

Using MALDI-MSI and proteomics analyses in matching HGSOC tissues at diagnosis and relapse, we identified proteins, including COL12A1, FUBP1, PLEC, SLC4A1, and TKT that could be novel therapeutic targets for chemoresistant HGSOC. Additional functional studies using in vitro and pre-clinical models are warranted to confirm the roles of these candidate proteins in ovarian cancer chemoresistance.

## Figures and Tables

**Table 1:** Kaplan–Meier plotter survival analysis of the relationship between mRNA expression of proteins of interest and HGSOC patient outcome. **(bold, *p* < 0.05).**

Gene Name	Progression-Free Survival (PFS)			Post-Progression Survival (PPS)			Overall Survival (OS)		
	HR	95% CI	*p*-Value	HR	95% CI	*p*-Value	HR	95% CI	*p*-Value
*COL12A1*	**1.76**	**1.38–2.24**	**3.7 × 10^−6^**	1.23	0.93–1.63	0.14	**1.38**	**1.05–1.83**	**0.022**
*FUBP1*	**1.43**	**1.22–1.68**	**1.1 × 10^−5^**	1.19	0.98–1.45	0.082	1.16	0.97–1.4	0.11
*PLEC*	1.15	0.97–1.36	0.1	**1.3**	**1.07–1.58**	**0.0071**	1.14	0.95–1.37	0.15
*SLC4A1*	0.83	0.65–1.07	0.15	0.87	0.65–1.15	0.32	0.84	0.63–1.12	0.23
*TKT*	**1.28**	**1.02–1.62**	**0.036**	1.26	0.94–1.68	0.12	**1.4**	**1.06–1.86**	**0.018**

## Data Availability

Data are available on request from the corresponding authors.

## Supplementary Materials

The supporting information can be downloaded at https://www.mdpi.com/article/10.3390/ijms26125893/s1.
