# Supplementary material for: Identification of Proteins Associated with Ovarian Cancer Chemotherapy Resistance Using MALDI-MSI
Source: Int J Mol Sci. 2025 Jun 19;26(12):5893. doi: 10.3390/ijms26125893 (PMC12193203; doi:10.3390/ijms26125893)
Supplement: Supplementary file 1 [file ijms-26-05893-s001.zip › Supp Material 11062025/Supplementary Figure.docx]

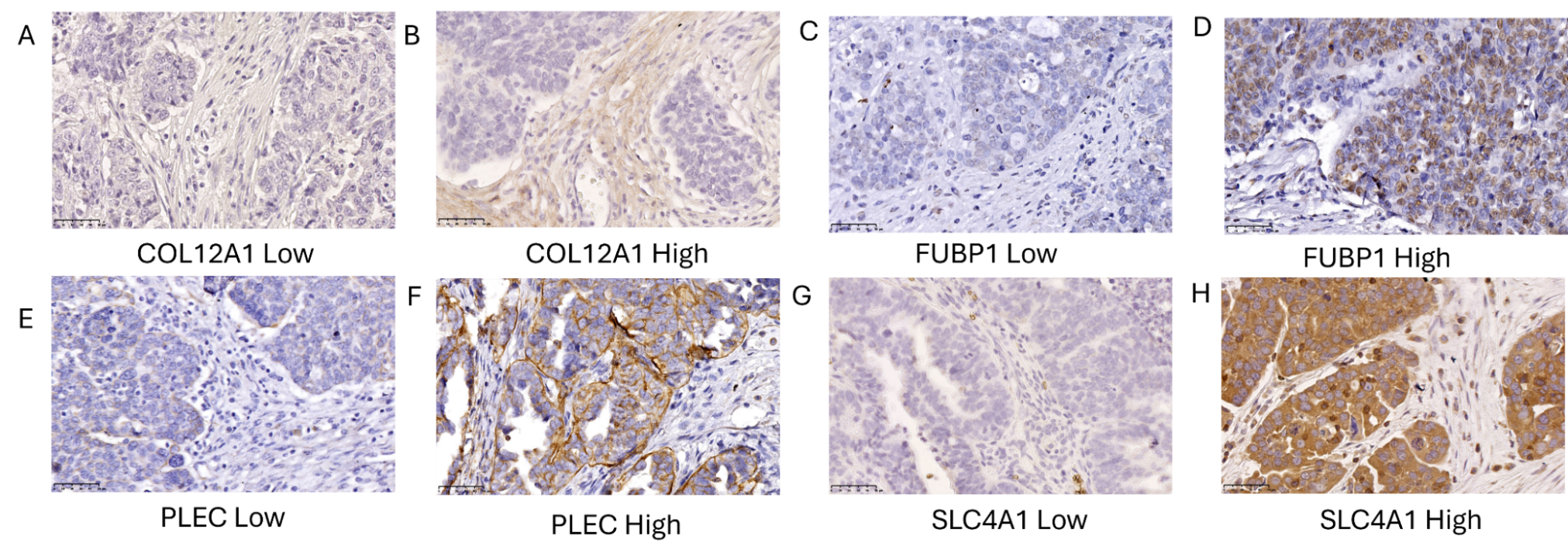


**Supplementary Figure S1:** Representative images of high and low immunostaining for COL12A1 (A-B), FUBP1 (C-D), PLEC (E-F), and SLC4A1 (G-H) in HGSOC tissues. Scale bar = 50µm.


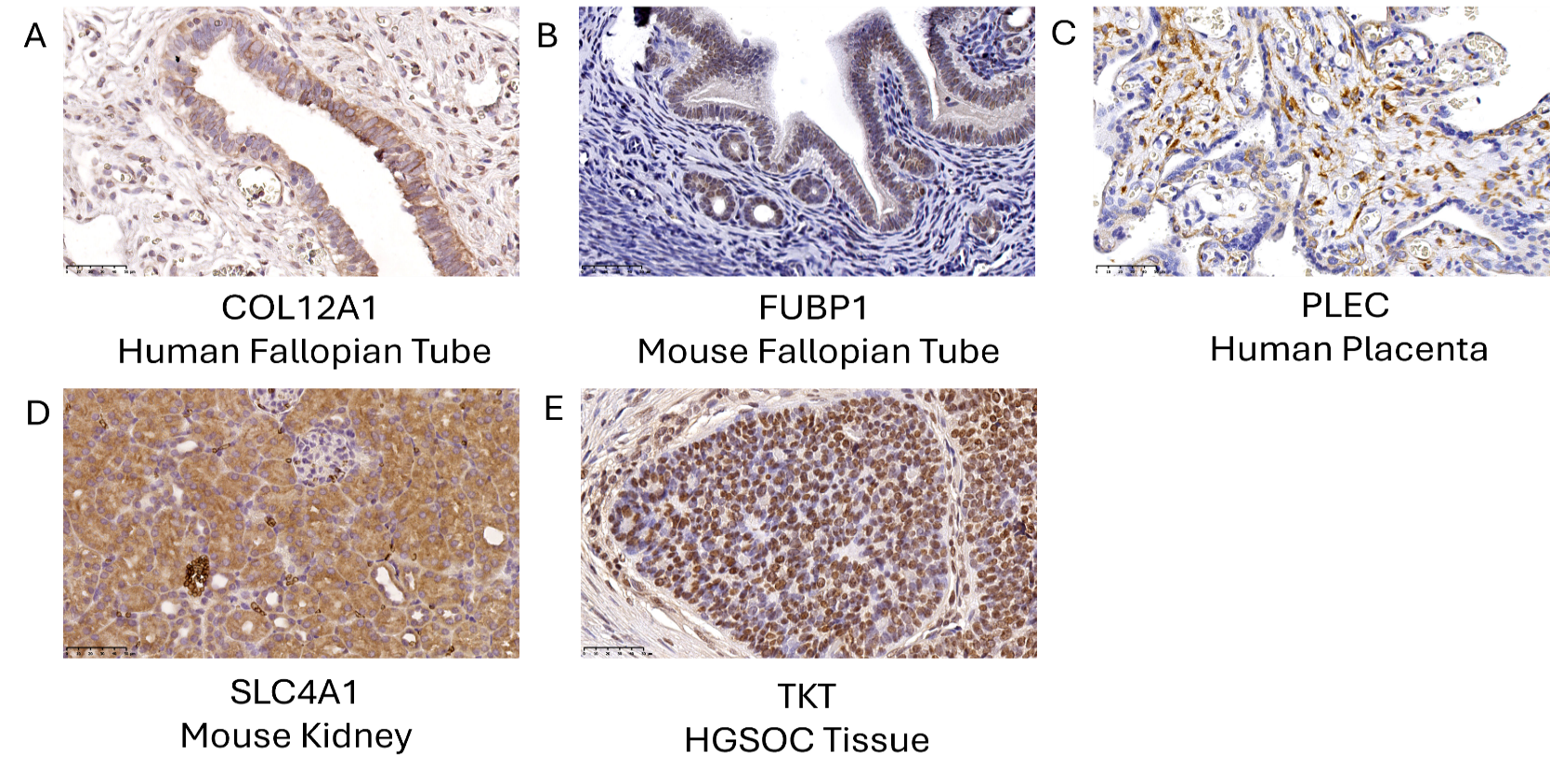


**Supplementary Figure S2:** Images of positive controls used for immunostaining. COL12A1 in human fallopian tube (A), FUBP1 in mouse fallopian tube (B), PLEC in human placenta (C), SLC4A1 in mouse kidney (D), and TKT in a HGSOC patient tissue (E). Scale bar = 50µm.

**
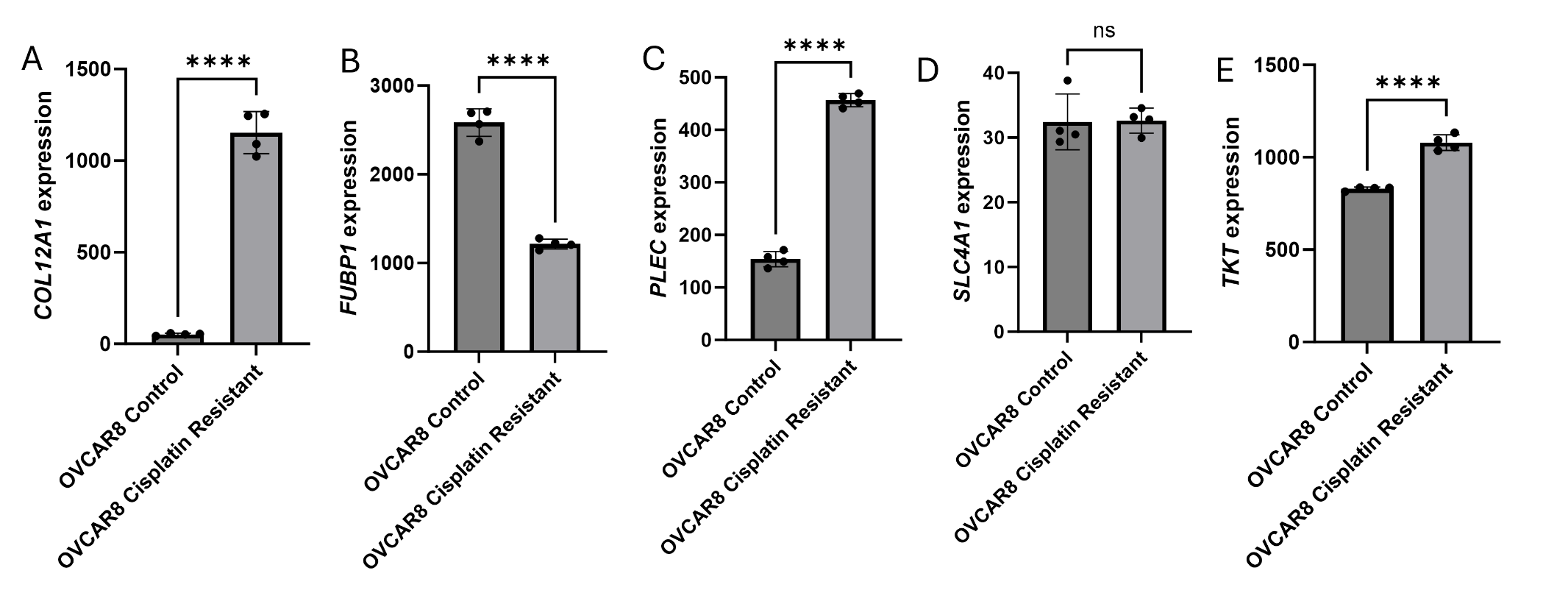
**

**Supplementary Figure S3:** mRNA expression of *COL12A1* (A), *FUBP1* (B), *PLEC* (C), *SLC4A1* (D), and *TKT* (E) in OVCAR-8 spheroids control (n=4) and OVCAR-8 cisplatin resistant spheroids (n=4). **** p<0.0001, ns=not significant. Unpaired t test. Data obtained from the GSE45553 database.
